# Supplementary material for: Exploring factors associated with dual tobacco smoking among people living with HIV receiving care at HIV outpatient clinics in Hanoi, Vietnam
Source: PLoS One. 2025 Nov 11;20(11):e0336112. doi: 10.1371/journal.pone.0336112 (PMC12604774; doi:10.1371/journal.pone.0336112)
Supplement: Attachment — Submitted filename: Rebuttal letter.docx [file pone.0336112.s003.docx]

**Rebuttal letter**

Dear Editor and Reviewers,

Thank you for reviewing our manuscripts. Below are our responses to both the journal requirement and reviewers’ comments.

---

**Journal requirement:**

1. I have ensured that the manuscript meets PLOS ONE's style requirements.
2. *Funding statement:*

“This study was supported by the National Cancer Institute under Award Number R01CA24481. THLH received a doctoral studentship at Sahlgrenska Academy to conduct the research. There was no additional external funding received for this study. The funders had no role in study design, data collection and analysis, decision to publish, or preparation of the manuscript.”

3. *Data policy*

The dataset underlying this study contains identifiable and highly sensitive health information (including HIV status). In accordance with determinations by the Institutional Review Boards at New York University and the Institute of Social and Medical Studies (ISMS), Hanoi, and applicable ethics approvals, the data cannot be placed in a public repository.

Non-author institutional contact for access requests. Qualified researchers may request access to a de-identified dataset for reproducibility purposes by contacting:

New York University – Institutional Review Board (IRB)

Scott Fisher, Director, Human Research Compliance

Email: srf291@nyu.edu

Web: NYU IRB Office

Requests must include a brief proposal outlining the intended use and analytic plan. Requests are reviewed independently by the NYU IRB for compliance with participant confidentiality and data-protection policies. If approved, requestors (and their institution) must sign a data-use agreement; additional local IRB/EC approvals may also be required. No author will adjudicate access decisions.

Location/citation of the dataset. The dataset is held by the New York University IRB and ISMS Ethics Committee and is not publicly archived. Please cite as:

Hoang T, et al. Exploring factors associated with dual tobacco smoking among people living with HIV receiving care at HIV outpatient clinics in Hanoi, Vietnam. Institute of Social and Medical Studies, Hanoi & New York University. 2020. Dataset available on approved request from the NYU IRB (contact above)

4. Thanh Hoang and Thanh Ha-Lan Hoang is the same author; I made sure the name is used consistently across the manuscript.
5. As above
6. I added the Ethical consideration sub-section under Methods that includes ethical approvals.
7. The manuscript has only one Figure; Figure 1’s caption was included. I removed Figure 2.
8. As above

**Reviewer’s comments:**

1. *Were questionnaires used to assess*

*-Smoking risk perceptions*

*-Intention to quit smoking*

*-Internalized smoking stigma*

*Validated in any way prior to use?*

In this revision, I cited in the manuscript the literature used to support the measurement of these variables.

**Risk perception** - Kaufman et al Measuring smoking cessation risk perception. NTR 2019 doi:10.1093/ntr/ntz213; https://apps.who.int/iris/bitstream/handle/10665/112836/9789241506946_eng.pdf

**Smoking stigma** questions were based on the Internalized Stigma of Smoking Inventory - Brown‐Johnson, C. G., Cataldo, J. K., Orozco, N., Lisha, N. E., Hickman III, N. J., & Prochaska, J. J. (2015). Validity and reliability of the internalized stigma of smoking inventory: An exploration of shame, isolation, and discrimination in smokers with mental health diagnoses. The American journal on addictions, 24(5), 410-418.

**Readiness to quit** - the specific citation we reference for the questions is a study that tested the model in the context of smoking -- DiClemente CC, Prochaska JO, Fairhurst SK, Velicer WF, Velasquez MM, Rossi JS. The process of smoking cessation: An analysis of precontemplation, contemplation, and preparation stages of change. Journal of Consulting.

2. *Regarding recruitment, please specify if participants received a compensation and if so, what form?*

The survey took approximately 45 minutes, and after its completion, the participant received an incentive of 2.5 USD. I added this in the Methods.

3. *Edits*

*In Data source section: Line 96 please define acronym NRT*

*In Measurement section: Line 129 ARV, Line 132 ART*

*In the Result section: Delete line 223, there is no Fig 2.*

I have addressed these issues.

---

I hope this revision meets the journal’s expectations. Please let me know if you have any questions.

Thank you very much.

Best Regards,

Thanh Ha-Lan Hoang, on behalf of co-authors
